# Mistrust in government and COVID-19 vaccination acceptance in Nigeria: investigating the indirect roles of attitudes towards vaccination

**DOI:** 10.1186/s42506-023-00129-5

**Published:** 2023-02-06

**Authors:** Babatola Olawa, Abiodun Lawal, Ikenna Odoh, Judith Azikiwe, Ayodeji Olawole, Emmanuel Odusina, Israel Ayodele, Olubukola Ajayi

**Affiliations:** 1 Department of Psychology, Federal University Oye-Ekiti, Oye-Ekiti, Nigeria; 2 University Medical Centre, Federal University Oye-Ekiti, Oye-Ekiti, Nigeria; 3 Department of Guidance and Counselling, Federal University Oye-Ekiti, Oye-Ekiti, Nigeria; 4 Department of Demography and Social Statistics, Federal University Oye-Ekiti, Oye-Ekiti, Nigeria; 5 Department of Psychology and Behavioural Studies, Ekiti State University, Ado-Ekiti, Nigeria

**Keywords:** Trust in government, Mistrust of vaccine benefits, Commercial profiteering, COVID-19 vaccination acceptance

## Abstract

**Background:**

Research shows that trust in government is associated with the acceptance of COVID-19 vaccination. However, there is no empirical evidence suggesting the pathway by which this association is formed. This study examines how dimensional attitudes towards vaccination explain the relationship between mistrust in government and COVID-19 vaccination acceptance.

**Methods:**

The study was an online cross-sectional survey involving 1026 adults (of which 58.9% are female) resident in Nigeria with a mean age of 26.09 (±8.46) years. Data were collected using structured questionnaires assessing the level of mistrust in government, dimensional attitudes towards vaccination, and acceptance to be vaccinated for COVID-19. Structural equation modeling was used to analyze data.

**Results:**

Results show that 56.8% of participants mistrust the government, while COVID-19 vaccination acceptance rate was 28.2%. Mistrust in government was significantly associated with low acceptance of COVID-19 vaccination. Furthermore, mistrust in the government was predictive of negative attitudes towards COVID-19 vaccination which include worries about unforeseen future effects of vaccines, mistrust of vaccine benefits (MVB), concerns about commercial profiteering (CCP), and preference for natural immunity. The outcomes of indirect effect analyses indicated that mistrust in government was associated with high mistrust in vaccine benefits (MVB) and increased concerns about commercial profiteering (CCP), which in turn lead to low acceptance of COVID-19 vaccination.

**Conclusions:**

Mistrust in the government was high and was coupled with low vaccination acceptance. It is important to initiate culturally relevant awareness programs aiming at combating false notions about COVID-19 vaccination such as MVB and CCP arising from mistrust in government.

## Introduction

Since their discovery more than 300 years ago, vaccines have been shown to be effective in curtailing the spread of diseases with a positive impact on human health and longevity [1]. With the troublesome success of SARS-CoV-2 and its variants at spreading the coronavirus disease 2019 (COVID-19), scientists have worked tirelessly at producing cutting-edge vaccines that are safe and that will reduce the high death rates recorded in different parts of world. As of 10th of July 2021, about 11 COVID-19 vaccines have been recommended by the World Health Organization including Pfizer/BioNTech, Moderna, Janssen, and Oxford/AstraZeneca [2]. Many of these vaccines have been proven to have durable effect in mitigating the risk of hospitalization and mortality; albeit with a waning efficacy as a result of immunity decline and occurrence of the delta variant [3].

Many nations have experienced high vaccine hesitancy rates despite the effectiveness of COVID-19 vaccines. For example, among Turkish respondents, it was reported that more than 65% showed reluctance in taking COVID-19 vaccines and especially those vaccines coming from foreign countries [4]. In a longitudinal survey of attitude towards vaccinations among 10 countries consisting of 8 European nations, South Africa and Australia, it was reported that only two countries (Belgium and Netherlands) showed positive attitudes over time [5]. In another survey of African countries, a 40% hesitancy rate was documented with 79% bothered about the unforeseen negative effects of vaccination [6]. However, a lower hesitancy rate of 20% was reported in one survey carried out among 15 African countries by the Africa Centers for Disease Control and Prevention [7]. This figure coincides with an 81% acceptance rate obtained in Nigeria [8]. Yet, some studies have reported hesitancy rates as high as 60–80% in other Nigerian samples [9–11].

In the 3Cs model of vaccine hesitancy, refusal or delay in vaccines’ acceptance despite their availability is determined by the 3Cs: confidence, complacency, and convenience [12]. Confidence refers to trust in the efficacy of vaccines together with their safety, the structure that offers them, and the interest of the policymakers who decide on the necessity of the vaccines. Complacency means the perception that the impact of the disease to be vaccinated against is low, and thus, vaccination is not considered as essential for prevention. Convenience indicates the extent of the availability, accessibility, and affordability of vaccines [13]. More important in the model is the role of confidence or trust in government and policymakers who make decisions about the relevance of vaccines to the populace. This was demonstrated by Trent et al. [14] where high trust in government promoted the willingness to receive COVOD-19 vaccines in some selected cities in Australia. Similar outcomes have been observed in Ethiopia [15], Ghana [16], and Belgium [17].

Although there is evidence linking trust in government and COVID-19 vaccination acceptance, we do not empirically know the mechanism underlying this association. The current study contributes to existing knowledge by proposing that trust in government influences rates of acceptance of COVID-19 vaccination via shaping of the citizen’s attitudes towards COVID-19 vaccines (ATCV). ATCV can be in the form of concerns that the vaccination program centers on commercial profiteering, anxiety about the unforeseen effect of the vaccine in the future, mistrust regarding the vaccine efficacy, and more preference for natural immunity rather than getting vaccinated [18].

The connections among trust in government, ATCV, and acceptance of COVID-19 vaccination are supported by the fairness model of trust which posits that perceptions of corruption and unfairness on the part of the government in the distribution of wealth and resources provoke negative attitudes of citizens towards governmental institutions, policies, and health programs [19, 20]. Given the possible unfavorable dispositions towards the government and its health institutions from the lack of public trust, citizens may cultivate negative attitudes towards the COVID-19 vaccination program together with the system that delivers it. Once negative ATCV is developed, there are higher odds that individuals will display high hesitant behavior based on the strong connection between attitudes and behavior as opined by the Theory of Reasoned Action [21].

Building on the possible associations among trust in government, ATCV, and COVID-19 vaccination acceptance, the following are hypothesized:

1. High levels of mistrust in government will significantly associate with low levels of COVID-19 vaccination acceptance.
2. High levels of mistrust in government will significantly associate with negative ATCV.
3. Negative ATCV will significantly associate with low levels of COVID-19 vaccination acceptance.
4. The relationship between trust in government and COVID-19 vaccination acceptance will be significantly accounted for by negative ATCV.

The outcomes of the study will provide significant contributions to the existing models of vaccination hesitancy which will be useful for implementers of public awareness programs geared towards promoting COVID-19 vaccination acceptance.

## Methods

### Study design

A cross-sectional design was used.

### Sample

The study’s sample consists of 1026 adults selected by convenience.

### Data collection

Data were collected via an online Google Form survey between April and August 2021 during the first phase of the COVID-19 vaccination program in Nigeria which commenced on 5th March 2021 [22]. The web link of the survey was sent to multiple online platforms such as students’ Facebook groups, WhatsApp groups, Twitter, and emails. The ethical approval to conduct the study was given by the Institutional Review Board of Federal University Oye-Ekiti. A statement about voluntary participation was included at the beginning of the google form. All participants who filled out the questionnaire were considered formerly consented to participating in the study.

### Measures

#### Acceptance of vaccination

The level of acceptance of COVID-19 vaccination was measured with a single item on a 5-point Likert scale ranging from (*1) strongly disagree* to (5) *strongly agree*. The item is worded as follows: *I would accept COVID-19 vaccine whenever I am approached to take it*. This study measures acceptance of the COVID-19 vaccination with a single item as in previous studies carried out within the Nigerian population (8, 9). Besides, a single-item measure has been recommended for one-dimensional constructs that can be easily understood such as asking whether someone would receive vaccination or not [23, 24]. High scores indicate an increased acceptance level of COVID-19 vaccination.

#### Attitude towards COVID-19 vaccines

It was assessed using the Vaccination Attitudes Examination (VAX) scale developed by Martin and Petrie [18]. The scale examines vaccination attitudes with 12 items on a 5-point scale ranging from *(1) strongly disagree* to *(5) strongly agree* along four dimensions, namely worries about unforeseen future effects (WFE), preference for natural immunity (PNI), mistrust of vaccine benefit (MVB), and concerns about commercial profiteering (CCP). Each of the dimensions of VAX is measured with three items. Sample items are as follows: *COVID-19 vaccines can cause unforeseen problems in people/children*, and *I will feel safe after being vaccinated for COVID-19*. The scale was shown to be a valid measure for identifying those “who are unlikely to get vaccinated and for identifying their strongest objections regarding vaccination” (18). The four-dimensional model of the VAX was subjected to confirmatory factor analyses and found to have acceptable model fit: *CFI* = 0.93, *RMSEA* = 0.06 (0.056, .072), and SRMR = 0.06, thus showing fitness to the current study data. The internal consistency coefficients for the four subscales are as follows: *WFE* = 0.59, *PNI* = 0.47, *MVB* = 0.86, and *CCP* = 0.64. High scores indicate negative attitudes towards COVID-19 vaccination.

#### Independent variable

- *Mistrust* in government was measured with a single item on a 7-point Likert scale ranging from (1) *a great deal* to (7) *not at all*. The item is worded as follows: *How much trust do you have in the Nigerian government today?* This measure is in line with the World Values Survey [25] and other previous studies [26, 27] that measured trust in the government with a single item. High scores reflect greater levels of mistrust in the government.
- *Sociodemographics/control variables*: Sociodemographic variables such as sex, age, marital status, education, occupation, religion, and geographical location were measured and used as control variables in model estimation.

### Statistical analyses

Descriptive statistics and bivariate relationships among study variables were performed using IBM SPSS software (20.0). Indirect effect analyses were carried out in IBM SPSS AMOS 28 using the maximum likelihood (ML) method. As displayed in Table 2, scores on mistrust in government deviated from moderate normality with a skewness score of −2.18. These scores were transformed using log_10_ transformation with reflection to achieve moderate normality. The standardized root-mean-square residual (SRMR), the comparative fit index (CFI), and the root mean square of approximation (RMSEA) were used to evaluate model fit. The recommended thresholds are as follows: CFI (> 0.90), SRMR, and RMSEA (< 0.08) [28, 29]. All sociodemographic variables with significant bivariate associations with focal variables were used as control variables in the model.

## Results

The complete sociodemographic data are presented in Table 1. The sample consists of 41.6% males and 58.9% females with a mean age of 26.09 (±8.46) years. More than 50% of the sample were 18–24 years old, while there were more students (67.4%) than the working class (29.1%). The majority of the sample were Christian (89.6%), unmarried (75.6%), lived in urban areas (74.5%), and studied up to the tertiary education level (92.7%).

**Table 1:** Sociodemographic characteristics of participants, Nigeria (2021)

Variables ***N*** = 1026	***n*** (%)
**Sex**	
Male	427 (41.6)
Female	599 (58.4)
**Age (years)**	
18–24	552 (53.8)
25–29	287 (28.0)
> 29	187 (18.2)
**Marital status**	
Unmarried	776 (75.6)
Married	250 (24.4)
**Education**	
Tertiary	951 (92.7)
Secondary	59 (5.8)
Primary	16 (1.6)
**Employment**	
Students	692 (67.4)
Public employment	151 (14.7)
Private employment	148 (14.4)
Unemployed	35 (3.4)
**Religion**	
Christianity	919 (89.6)
Islam	91 (8.9)
Others	16 (1.6)
**Residence**	
Rural	262 (25.5)
Urban	764 (74.5)

### Level of trust in government and acceptance rate of COVID-19 vaccines

The levels of trust in government and acceptance of COVID-19 vaccination are shown in Table 2. Results showed that 56.8% of participants do not trust the Nigerian government, while 27.6% had very little trust. Only 5.4% expressed much to a great deal of trust in government. The acceptance rate for COVID-19 vaccination was 28.2%, while 26% were undecided on whether to take the vaccine or not.

**Table 2:** Levels of trust in government and acceptance of COVID-19 vaccination, Nigeria (2021)

Variables ***N*** = 1026	***n*** (%)
**Trust in government**	
Not at all	583 (56.8)
Very little	283 (27.6)
Little	65 (6.3)
Somewhat	40 (3.9)
Much	31 (3.0)
Very much	9 (0.9)
A great deal	15 (1.5)
**Vaccination acceptance**	
Strongly disagree	297 (28.9)
Disagree	173 (16.9)
Undecided	267 (26.0)
Agree	196 (19.1)
Strongly agree	93 (9.1)

### Bivariate associations among focal variables

Table 3 shows the bivariate associations among focal variables and sociodemographic factors. Mistrust in the government was found to be positively associated with all attitude dimensions towards vaccination which are worries about the anticipated future effect of COVID-19 vaccines (*r* = 0.18, *p* < 0.001), misgivings regarding commercial profiteering (*r* = 0.25, *p* < .001), mistrust concerning the benefit of vaccines (*r* = 0.16, *p* < 0.001), and preference for natural immunity (*r* = 0.07, *p* = 0.03). A significant and negative relationship was found between mistrust in government and acceptance of vaccines (*r* = −0.20, *p* < 0.001). Attitudes towards vaccination were all negatively related to acceptance of COVID-19 vaccination [MVB (*r* = −0.56, *p* < 0.001); CCP (*r* = −0.23, *p* < 0 .001); WFE (*r* = −0.11, *p* = 0.001); and PNI (*r* = −0.11, *p* < 0.001)].

**Table 3:** Bivariate correlations and descriptive statistics

*N* = 538	1	2	3	4	5	6	7	8	9	10	11	12	13
Sex (1)													
Age (2)	−0.13^a^												
Marital status (3)	−.09^b^	0.64^a^											
Occupation (4)	0.11^a^	−0.61^a^	−0.66										
Education (5)	.03	−.06	−0.10^b^	0.13^a^									
Religion (6)	.05	−.09	−0.12^b^	.04	.08^b^								
Residence (7)	.05	.03	.04	−.06^b^	.04	−.06							
Mistrust in the govt. (8)	.01	−.05	−.01	.03	.06^b^	0.10^a^	−.03						
Future effect of vaccine (9)	−.07^b^	−.03	−.06	−.02	.04	0.10^a^	0.11^a^	0.18^a^					
Vaccine mistrust (10)	.06	.09^a^	.09^a^	−.08^b^	−.01	.08	−.04	0.16^a^	0.10^a^				
Natural immunity (11)	−.02	.04	.05	−.07	−.01	.004	−.04	.07^b^	0.20^a^	0.18^a^			
Commercial profiteering (12)	−.02	−.06	−.09	.03	.04	.04	−.004	0.25^a^	0.40^a^	0.25^a^	0.38^a^		
Acceptance (13)	−.07	−.003	.01	−.01	−.07	−.07^b^	−.01	−0.20^a^	−0.11^a^	−0.56^a^	−0.11^a^	−0.23^a^	
Mean								6.23	10.74	9.53	8.76	9.84	2.62
SD								1.23	2.62	3.32	2.58	2.90	1.32
Skewness								−2.18	−0.60	.01	0.13	−0.16	0.19
Kurtosis								5.03	.02	−0.96	−0.45	−0.54	−1.14

^a^Correlation is significant at the 0.01 level (2-tailed). ^b^Correlation is significant at the 0.05 level (2-tailed). Gender (0 = male, 1 = female); marital status (0 = married, 1 = unmarried); education (0 = others, 1 = tertiary education); occupation (0 = others, 1 = students); religion (0 = others, 1 = Christianity); residence (0 = rural, 1 = urban)

### Path model

**Fig. 1:**
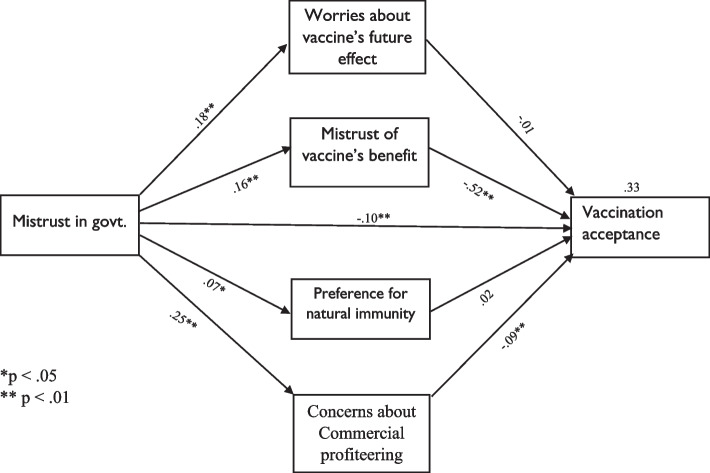
Path model

Figure 1 shows the indirect roles of attitudes towards COVID-19 vaccination in the association between mistrust in government and vaccine acceptance.

Table 4 shows that the model has an acceptable model fit, *χ*^2^ (45) = 353.49, *p* < 0.01; *CFI* = 0.99; *RMSEA* = 0.03 (90% *CI* = (0.01, 0.04); and SRMR = 0.03. Increase in mistrust in government is associated with lower acceptance of vaccination (*β* = −0.10, *p* < 0.001). Also, an increase in mistrust in government was related to an increase in CCP (*β* = 0.25, *p* < 0.001), WFE (*β* = 0.18, *p* < 0.001), MVB (*r* = 0.16, *p* < 0.001), and PNI (*β* = 0.07, *p* = 0.03). Moreover, greater levels of CCP (*β* = −0.09, *p* = 0.005) and MVB (*β* = −0.52, *p* < 0.001) were associated with a lower acceptance of vaccination, while WFE (*β* = −0.01, *p* = 0.86) and PNI (*β* = 0.02, *p* =0.47) were not significant on vaccination acceptance.

**Table 4:** Unstandardized indirect effects and model fitness

	10,000-sample bootstrapping	Model fitness				
		*χ*^2^ (*df*)	*p*-value	RMSEA (90% *CI*)	CFI	SRMR
*Indirect effect*						
Mistrust in govt. → WFE → acceptance	−0.01 (−.06, −0.07)					
Mistrust in govt. → MVB → acceptance	**−0.48 (0.29, 0.69)**					
Mistrust in govt. → PNI → acceptance	0.01 (**−**0.04, 0.01)	45.78 (27)	0.01	0.03 (0.01, 0.04)	0.99	0.03
Mistrust in govt. → CCP → acceptance	**−0.12 (0.03, 0.23)**					

*WFE* Worries about unforeseen future effects, *PNI* Preference for natural immunity, *MVB* Mistrust of vaccine benefit, *CCP*, Concerns about commercial profiteering. Bold denotes significance

### Indirect effect analyses

In a structural equation modeling framework, confirming an indirect effect is dependent on detecting significance for both total and indirect effects [30]. The standardized total effect of mistrust in government on acceptance of COVID-19 vaccine was significant (*β* = −0.20, *p* < 0.001). The 95% bias-corrected confidence intervals (CI) for unstandardized indirect effect with 10,000 bootstrap samples are displayed in Table 4. Results showed that mistrust in vaccine benefit (*B* = −0.48 [−0.29, −0.69]) and concerns regarding commercial profiteering (*B* = −0.12 [−0.03, −0.23]) significantly accounted for the negative relationship between mistrust in government and vaccination acceptance since the CIs did not pass through zero. However, worries about the future effect of vaccination (*B* = −0.01 [−0.06, 0.07]) and preference for natural immunity (*B* = 0.01 [−0.04, 0.01]) did not account for the association between mistrust in government and vaccination acceptance as the CIs passed through zero.

## Discussion

Previous works showed that trust in government is associated with the acceptance rate of COVID-19 vaccination [14, 16]. However, there is no empirical evidence on the pathway by which this association is formed. Based on the 3C’s model of vaccine hesitancy, fairness model of trust, and the Theory of Reasoned Action, the present study proposes that trust in government impacts COVID-19 vaccination acceptance via shaping peoples’ attitude towards vaccination.

Results of direct relationships indicate that all study variables are associated in the direction anticipated. Mistrust in government was positively associated with all dimensions of negative attitudes towards COVID-19 vaccination. Mistrust in government had greater influence on concerns about commercial profiteering than other dimensions. These relationships underscore how a lack of trust in government can influence people’s perception of COVID-19 vaccines. If there is a deficiency in the levels of trust in government, it may predispose citizens to negative attitudes towards its healthcare programs even if they are of greatest benefits. Lack of trust or mistrust can make people underestimate the risks of the disease to be vaccinated against, undervalue the effectiveness of the vaccine, and nurture various negative beliefs about the vaccination program [31], including the notion that the vaccination program is geared towards commercial purposes and diversion of public funds [32].

Mistrust in government was also found to be negatively related to acceptance of COVID-19 vaccines. That is, the more individuals do not trust the government, the less their willingness to accept the vaccines is. This is not surprising given the high level of mistrust in government and the low vaccine acceptance rate found in this study. This outcome corroborates recent studies that established a link between trust in government and acceptance of COVID-19 vaccines [16, 17]. In addition, a relationship was found between attitudes towards COVID-19 vaccination (ATCV) and its acceptance. Specifically, only two dimensions of ATCV — concerns about commercial profiteering (CCP) and mistrust of vaccine benefits (MVB) — were found to be associated with COVID-19 acceptance with MVB exerting a greater influence. These imply that the belief that vaccination programs are aimed at enriching vaccine manufacturers and the government, together with a lack of trust in the efficacy of COVID-19 vaccine, could be fundamental to high COVID-19 hesitancy rate among the population. These findings agree with a qualitative study conducted in Nigeria, in which participants erroneously believed that COVID-19 is not real, and as such, the vaccine is fake with the government officials out to misuse public funds for their benefit [32].

More importantly, results show that the negative association between mistrust in government and COVID-19 vaccination acceptance can be explained by the MVB and the CCP. Specifically, greater mistrust in government produces more doubts regarding the benefits of COVID-19 vaccines, which in turn leads to a low acceptance rate of vaccines. Similarly, higher levels of mistrust in government are associated with more concerns about commercial profiteering, which in turn leads to a low acceptance rate of vaccines. These findings are important and novel. They empirically suggest that a pathway to increasing COVID-19 vaccination acceptance rate is by promoting trust in government, which will in turn reshape citizens’ belief about the efficacy of the COVID-19 vaccine and counter the notion about commercial profiteering. These results confirm the fairness model of trust which assumes that perception of corruption and unfairness by the government in the distribution of wealth and resources can generate citizens’ mistrust, which will then affect their beliefs, attitudes, and behaviors towards newly introduced social and health programs [19].

### Limitations of the study

Although this study has some important contributions to knowledge, it is important to point out its limitations. The use of the cross-sectional approach limits the ability to draw causal relationships among study variables. At best, the associations established among variables are correlational. In addition, the majority of study sample are Christians and individuals who studied up to the tertiary education level. Generalizing findings to people in other religions and those with lower education or without formal education may be limited. Future studies considering the replication of this work may improve on these limitations.

## Conclusion

Mistrust in the benefits of COVID-19 vaccines and concerns about commercial profiteering accounted for the relationship between mistrust in government and the low acceptance rate of COVID-19 vaccination. Mistrust in government can lead to the negative notion of commercial profiteering and suspicions regarding the efficacy of vaccines and eventually high vaccine hesitancy. To increase its acceptance rate, it is important to initiate programs that will increase peoples’ trust in government alongside other awareness programs aimed at combating false beliefs about COVID-19 vaccination. Programs to improve public trust may involve an advocacy on the transparency of the government especially in relation to the curtailment of the COVID-19 pandemic.

## Data Availability

Available from the corresponding author on reasonable request.

## Abbreviations

COVID-19: Coronavirus disease of 2019
ATCV: Attitudes towards COVID-19 vaccines
VAX: Vaccination attitudes examination
WFE: Worries about unforeseen future effects
PNI: Preference for natural immunity
MVB: Mistrust of vaccine benefit
CCP: Concerns about commercial profiteering
ML: Maximum likelihood
IBM SPSS: International Business Machines Statistical Package for the Social Sciences
SRMR: Standardized root-mean-square residual
CFI: Comparative fit index
RMSEA: Root-mean-square error of approximation

## References

[1] Plotkin S (2014). History of vaccination. PNAS.

[2] World Health Organization (2022). COVID-19 vaccines with WHO emergency use listing.

[3] Lin DY, Gu Y, Wheeler B, Young H, Holloway S, Sunny SK (2022). Effectiveness of Covid-19 vaccines over a 9-month period in North Carolina. N Engl J Med.

[4] Yigit M, Ozkaya-Parlakay A, Senel E (2021). Evaluation of COVID-19 vaccine refusal in parents. Pediatr Infect Dis J.

[5] Greyling T, Rossouw S (2022). Positive attitudes towards COVID-19 vaccines: a cross-country analysis. PloS One.

[6] Anjorin AA, Odetokun IA, Abioye AI, Elnadi H, Umoren MV, Damaris BF (2021). Will Africans take COVID-19 vaccination?. PloS One..

[7] African Center for Disease Control (2020). majority of Africans would take a safe and effective COVID-19 vaccine.

[8] Adedeji-Adenola H, Olugbake OA, Adeosun SA (2022). Factors influencing COVID-19 vaccine uptake among adults in Nigeria. PloS One.

[9] Uzochukwu IC, Eleje GU, Nwankwo CH, Chukwuma GO, Uzuke CA, Uzochukwu CE, et al. COVID-19 vaccine hesitancy among staff and students in a Nigerian tertiary educational institution. Ther Adv Infect Dis. 2021. 10.1177/20499361211054923. 10.1177/20499361211054923 PMC8564127 34745608

[10] Mustapha T, Khubchandani J, Biswas N (2021). COVID-19 vaccination hesitancy in students and trainees of healthcare professions: a global assessment and call for action. Brain Behav Immun.

[11] Olu-Abiodun O, Abiodun O, Okafor N (2022). COVID-19 vaccination in Nigeria: a rapid review of vaccine acceptance rate and the associated factors. PloS One.

[12] SAGE Working Group (2014). report of the SAGE Working Group on vaccine hesitancy.

[13] Verhoest, de BS, Glavina M (2020). Trust & vaccination.

[14] Trent M, Seale H, Chughtai AA, Salmon D, MacIntyre CR (2022). Trust in government, intention to vaccinate and COVID-19 vaccine hesitancy: a comparative survey of five large cities in the United States, United Kingdom, and Australia. Vaccine.

[15] Strupat C, Shigute Z, Bedi AS, Rieger M (2022). Willingness to take COVID-19 vaccination in low-income countries: evidence from Ethiopia. PloS One.

[16] Amo-Adjei J, Nurzhynska A, Essuman R, Lohiniva A (2022). Trust and willingness towards COVID-19 vaccine uptake: a mixed-method study in Ghana, 2021. Arch Public Health.

[17] Wynen J, Op de Beeck S, Verhoest K, Glavina M, Six F, Van Damme P, et al. Taking a COVID-19 vaccine or not? Do trust in government and trust in experts help us to understand vaccination intention? Adm Soc. 2022. 10.1177/00953997211073459.

[18] Martin LR, Petrie KJ (2017). Understanding the dimensions of anti-vaccination attitudes: the vaccination attitudes examination (VAX) scale. Ann Behav Med.

[19] Addai I, Opoku-Agyeman C, Ghartey HT (2013). An exploratory study of religion and trust in Ghana. Soc Indic Res.

[20] Sapsford R, Tsourapas G, Abbott P, Teti A (2019). Corruption, trust, inclusion and cohesion in North Africa and the Middle East. Appl Res Qual Life.

[21] Fishbein M, Ajzen I (2010). Predicting and changing behavior: the reasoned action approach.

[22] Al-Mustapha AI, Oyewo M, Olugbon AS, Elelu N (2021). A mix and match approach to COVID-19 vaccinations: does Nigeria have a choice?. Front Public Health..

[23] Fuchs C, Diamantopoulos A (2009). Using single-item measures for construct measurement in management research. Bus Manag Rev.

[24] Sauro J (2018). Is a single item enough to measure a construct?.

[25] Inglehart R, Haerpfer C, Moreno A, Welzel C, Kizilova K, Diez-Medrano J (2014). World Values Survey: Round Six - Country-Pooled Datafile version.

[26] Alford JR, Hibbing JR, Theiss-Morse E (2001). We’re all in this together: the decline of trust in government, 1958-1996. What is it about government that Americans dislike?.

[27] Hibbing JR, Smith JT (2004). Is it the middle that is frustrated? Americans’ ideological positions and governmental trust. Am Politics Res.

[28] Hu L-t, Bentler PM (1999). Cutoff criteria for fit indexes in covariance structure analysis: conventional criteria versus new alternatives. Struct Equ Model.

[29] Kline RB (2011). Principles and practice of structural equation modeling.

[30] Preacher KJ, Hayes AF (2004). SPSS and SAS procedures for estimating indirect effects in simple mediation models. Behav Res Meth Instrum Comput.

[31] Larson HJ, Jarrett C, Eckersberger E, Smith DM, Paterson P (2014). Understanding vaccine hesitancy around vaccines and vaccination from a global perspective: a systematic review of published literature, 2007-2012. Vaccine.

[32] Wonodi C, Obi-Jeff C, Adewumi F, Keluo-Udeke SC, Gur-Arie R, Krubiner C (2022). Conspiracy theories and misinformation about COVID-19 in Nigeria: implications for vaccine demand generation communications. Vaccine.

